# QTLs Regulating the Contents of Antioxidants, Phenolics, and Flavonoids in Soybean Seeds Share a Common Genomic Region

**DOI:** 10.3389/fpls.2016.00854

**Published:** 2016-06-14

**Authors:** Man-Wah Li, Nacira B. Muñoz, Chi-Fai Wong, Fuk-Ling Wong, Kwong-Sen Wong, Johanna Wing-Hang Wong, Xinpeng Qi, Kwan-Pok Li, Ming-Sin Ng, Hon-Ming Lam

**Affiliations:** ^1^Centre for Soybean Research of the Partner State Key Laboratory of Agrobiotechnology and School of Life Sciences, The Chinese University of Hong KongHong Kong, China; ^2^Instituto de Fisiología y Recursos Genéticos Vegetales, Centro de Investigaciones Agropecuarias–INTACórdoba, Argentina; ^3^Cátedra de Fisiología Vegetal, Facultad de Ciencias Exactas Físicas y Naturales, Universidad Nacional de CórdobaCórdoba, Argentina

**Keywords:** antioxidants, flavonoids, genome, human selection, MATE transporter, phenolics, quantitative trait loci, soybean

## Abstract

Soybean seeds are a rich source of phenolic compounds, especially isoflavonoids, which are important nutraceuticals. Our study using 14 wild- and 16 cultivated-soybean accessions shows that seeds from cultivated soybeans generally contain lower total antioxidants compared to their wild counterparts, likely an unintended consequence of domestication or human selection. Using a recombinant inbred population resulting from a wild and a cultivated soybean parent and a bin map approach, we have identified an overlapping genomic region containing major quantitative trait loci (QTLs) that regulate the seed contents of total antioxidants, phenolics, and flavonoids. The QTL for seed antioxidant content contains 14 annotated genes based on the Williams 82 reference genome (Gmax1.01). None of these genes encodes functions that are related to the phenylpropanoid pathway of soybean. However, we found three putative Multidrug And Toxic Compound Extrusion (MATE) transporter genes within this QTL and one adjacent to it (*GmMATE1-4*). Moreover, we have identified non-synonymous changes between *GmMATE1* and *GmMATE2*, and that *GmMATE3* encodes an antisense transcript that expresses in pods. Whether the polymorphisms in GmMATE proteins are major determinants of the antioxidant contents, or whether the antisense transcripts of *GmMATE3* play important regulatory roles, awaits further functional investigations.

## Introduction

Plant secondary metabolites can be classified into three major groups: phenolics, terpenoids, and alkaloids (Wink, 2010). Phenolic metabolites in plants are derived from aromatic amino acids via the phenylpropanoid pathway, and there are two major types: flavonoids and non-flavonoids. The term “flavonoids” could be confusing in the literature. Strictly speaking, they are compounds with the 2-phenylchromen-4-one backbone while “isoflavonoids” are compounds with the 3-phenylchromen-4-one backbone. In this work, we extend our use of the term “flavonoids” to also include isoflavonoids.

Soybean seeds are a rich source of phenolic compounds, especially isoflavonoids (Malencic et al., 2007). This characteristic has a significant impact on nutrition and health since phenolic compounds extracted from soybean seeds exhibit anticancer activities on a wide-range of cancer cell lines including breast, colorectal, hepatocellular leukemia, tongue, ovarian, prostate, and gastric cancers (Xu et al., 2010; Xu and Chang, 2011, 2012; Dong et al., 2012). On the other hand, the mechanism of phenolics accumulation in soybean seeds is not completely understood. Efforts have also been made to study the possible relationships among antioxidant activities, phenolic compound contents, and flavonoid contents in cultivated soybean, especially those with black seed coat (Phommalath et al., 2014), although antioxidant activities do not seem to be directly associated with the seed coat color (Cho et al., 2013; Qi et al., 2014).

Next-generation sequencing-based studies have provided solid evidence to support that wild soybeans are important genetic resources for crop improvement (Kim et al., 2010; Lam et al., 2010; Zhou et al., 2015). Using a recombinant inbred (RI) population built from the cross between a wild and a cultivated soybean parent, our previous study has identified a quantitative trait locus (QTL) regulating seed antioxidant contents on chromosome 19 (Qi et al., 2014). This QTL does not overlap with the QTL controlling seed coat color and anthocyanin content (Qi et al., 2014), suggesting that the high seed antioxidant activities in wild soybeans are not a direct result of high anthocyanin contents in their seed coats. Moreover, this QTL is also distinct from the genistein and total isoflavone QTL region on chromosome 19, which contains a phenylalanine-ammonia lyase (PAL)-encoding gene (Gutierrez-Gonzalez et al., 2010b). PAL catalyzes the committing step in the phenolic and flavonoid synthesizing phenylpropanoid pathway (Reinprecht et al., 2013).

In this study, we investigated the relationships between total seed antioxidant contents, seed phenolic contents, and seed flavonoid contents in hulled seeds of the RI population, and found one genomic region covering all three QTLs. Upon further analyses to refine this genomic region, several *Multidrug And Toxic Compound Extrusion* (*MATE*) genes controlling the transportation of secondary compounds were identified therein.

## Materials and Methods

### Plant Materials, Growth Conditions and Reference Genomes

Fourteen cultivated and 16 wild soybean germplasms were taken from a previously re-sequenced collection (Supplementary Table S1) (Lam et al., 2010). A RI population originating from reciprocal crosses of W05 (wild) and C08 (cultivated) was adopted from a previous study (Qi et al., 2014). In brief, lines were propagated on a single-seed descendant basis from F2 to F7. All seeds of each line were bulked up since F8. Seeds for analyses were collected from open fields (22°25′7′′ N, 114°12′26′′ E) and (38°35′0 ′′ N, 116°48′0′′E) from 2010 to 2012.

### Measurement of Antioxidant Contents, Total Phenolic and Total Flavonoid Contents in Seeds

For hulled seed measurements, seeds were hulled by hand and ground with pestle and mortar. Seed antioxidant contents were determined with the trolox equivalent antioxidant capacity (TEAC) assay (Miller et al., 1996). In brief, antioxidants from 0.5 g of powdered seeds were extracted with 10 ml 0.1N HCl in 80% methanol (∼pH1.1) overnight in the dark with shaking. After centrifugation, the supernatant was collected and dried with a rotary evaporator and re-constituted with 1 ml of deionized water. Anti-oxidation activities were determined by measuring the decrease in absorbance at 734 nm upon the reduction of 200 μl ABTS^+^ radicals by 10 μl of samples. The quantitative measurement of antioxidants in samples was made with reference to the anti-oxidation activities of trolox standards.

Phenolics and flavonoids were extracted from powdered hulled seeds. One hundred and fifty milligrams of the seed powder were weighed and mixed with 1 ml of 80% methanol. Extraction was performed with shaking (200 rpm) at 28°C overnight in the dark. Samples were then centrifuged at 14,000 rpm and the supernatant was transferred to a clean tube. The same extract was used for total phenolics and total flavonoids measurements.

Total flavonoids were determined following a previously described protocol (Zhu et al., 2010) with some modifications. Three hundred microliters of the sample extract was mixed with 300 μl of deionized water in a clean tube. Then 30 μl of 5% NaNO_2_ was added to the sample and incubated at room temperature for 5 min. After this first incubation, 60 μl of 10% AlCl_3_ was added to the mixture and incubated for 5 min at room temperature. Finally, 200 μl of 1 M NaOH was added and mixed. The absorbance at 500 nm of each sample was read using Synergy H1 Multi-Mode Reader (BioTek Instruments, Inc.). The quantitative measurement of flavonoids in each sample was made using a standard curve prepared with quercetin.

Total phenolics were determined following a previously described protocol (Ainsworth and Gillespie, 2007). Briefly, 20 μl of the sample extract was diluted with 20 μl phosphate buffered saline (PBS) and mixed with 100 μl of 10-fold diluted Folin–Ciocalteu reagent. Then 80 μl of 7.5% Na_2_CO_3_ was added, vortexed and incubated for 30 min in the dark. After incubation, the sample was centrifuged at 14,000 rpm. The absorbance of the supernatant was measured at 765 nm with Synergy H1 Multi-Mode Reader. The quantitative measurement of phenolics in the sample was made using a standard curve prepared with gallic acid.

### QTL Mapping

QTLs were identified with a bin map published (Qi et al., 2014) using QTL Cartographer^1^. In brief, composite interval mapping with a 10-centimorgan (cM) scanning window and a 0.5-cM walking step were adopted. The LOD cut-off was determined with 1,000 times permutation with *p* < 0.05. The QTL boundary was determined with a 1.5-drop of the LOD score from the highest score (Qi et al., 2014).

### Phylogenetic Studies

A Maximum Likelihood tree was built using MEGA (version 6) with 1,000 bootstrap replications for the phylogeny test, the Jones–Taylor–Thornton (JTT) substitution model, a complete deletion of gaps/missing data, a neighbor-joining initial tree and the Nearest–Neighbor–Interchange (NNI) ML Heuristic Method.

### Gene Expression Studies

Total RNA was extracted from primary leaves and roots at the seedling stage and from pods at five different pod developmental stages. For the seedling stage, soybean seeds were germinated in a greenhouse on vermiculite. Seven-day-old seedlings were transferred to a hydroponic system with one-strength Hoagland’s solution. When the primary leaves were fully opened, samples were collected and immediately frozen in liquid nitrogen. For pods, soybean seeds were germinated and grown in a greenhouse. Pods were harvested at 7, 14, 28, 40, and 60 days after flowering (DAF). Whole 7-DAF pods and pod shells or the developing seeds of the remaining time points were subjected to total RNA extraction.

RT-qPCR was conducted with One-Step SYBR PrimeScript RT-PCR Kit I (RR086A, Takara Bio Inc.) on a CFX96 Touch Real-Time PCR Detection System (BioRad, CA) according to manufacturers’ instructions. In brief, 50 ng DNaseI-treated total RNA was added to each 15 μl reaction containing 0.4 μM of each primer, 1X Buffer 4 and 0.6 μl of PrimeScript One step enzyme mix. The reaction began with reverse transcription at 42°C for 5 min, followed by heat inactivation at 95°C for 10 s, followed by 40 cycles of a denaturation step at 95°C for 5 s and an annealing/extension step at 55°C for 30 s. A dissociation curve was obtained, starting at 65°C with a 0.5°C increment for 5 s at each temperature until 95°C was reached. For strand-specific analyses, only one primer was added to the reaction before the reverse transcription step. The other primer was added after the reverse transcriptase was inactivated for 1 min at 95°C. The primer information for RT-qPCR can be found in Supplementary Table S3. The housekeeping genes *ELF1b* and *Bic-C2* (Yim et al., 2015) were used to normalize RNA input in the reaction. The relative gene expression level was determined by the 2^-ΔΔCT^ method (Livak and Schmittgen, 2001).

### Amplification and Sequence Analyses of the *MATE* Genes

The coding sequences of *GmMATE1*, *GmMATE2*, and *GmMATE4* were amplified from oligo-dT reverse-transcribed cDNAs prepared from mixed pods of different ages. Each 25-μl reaction contained 1X Phusion HF buffer, 20 ng cDNA, 0.5 μM of each primer, 0.2 mM dNTP mix and 0.5 U of Phusion polymerase (NEB, M0530). The thermocycler was programmed as follows: 98°C for 2 min, 40 cycles of 98°C for 15 s, 60°C for 10 s, and 72°C for 1 min, followed by 72°C for 10 min for the final extension. The primer information can be found in Supplementary Table S3. Sequencing of the PCR products was done using a commercial service (Macrogen Inc.) with gene-specific primers. The resulting amino acid sequences were aligned with BioEdit (Hall, 1999). *GmMATE3* was amplified from oligo-dT reverse-transcribed cDNAs from primary leaves, roots, flowers, 14-DAF pods, and 21-DAF and 28-DAF pod shells. The sense/antisense transcripts of *GmMATE3* were confirmed by PCR using cDNA reverse-transcribed with either the forward or the reverse primer of *GmMATE3*. The transmembrane topologies of GmMATE1, GmMATE2, and AtFRD3 were generated with Protter (Omasits et al., 2014).

### Statistical Analyses

*k*-Means clustering analyses, correlation analyses of phenotypic data and Student’s *t*-test of gene expression were done with R version 3.1.3. Normality tests were done with the Shapiro-Wilk test using IBM SPSS Statistics for Windows, Version 22.0 (IBM Corp, NY, USA). The transformation of data was done according to established procedure (Templeton, 2011).

## Results

### Identification of QTLs Regulating Seed Antioxidant Contents, Seed Phenolic Contents, and Seed Flavonoid Contents Using Hulled Seeds of a Recombinant Inbred Population

**FIGURE 1:**
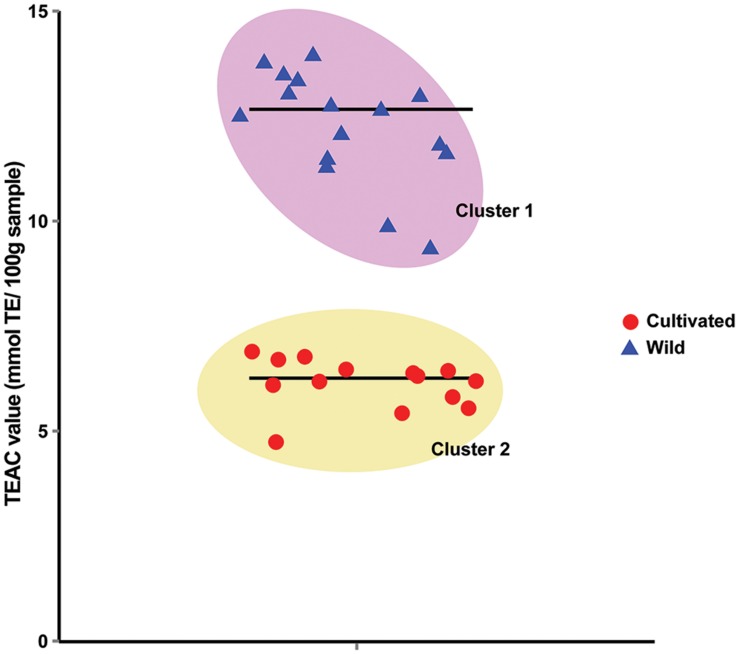
***k*-Mean clustering of the trolox equivalent antioxidant capacity (TEAC) values of 30 soybean germplasms**. The mean TEAC values of 3 biological repeats of 30 soybean germplasms were clustered with *k* = 2. Horizontal lines in the graph mark the mean values of the corresponding clusters.

To test the hypothesis that wild soybeans have higher seed antioxidant activities, we measured total seed antioxidant contents from 14 cultivated and 16 wild soybean accessions using the trolox equivalent antioxidant capacity (TEAC) assay. The TEAC values of the samples ranged from 4.75 to 13.79 mmol Trolox Equivalents (TE) 100 g^-1^ sample. *k*-means clustering with *k* = 2 using the TEAC values successfully separated the 30 germplasms into two clusters, each containing only either wild or cultivated soybeans (**Figure 1**). Cluster means were 12.33 and 6.14 mmol TE 100 g^-1^ sample for cluster 1 (wild soybeans) and cluster 2 (cultivated soybeans), respectively. This suggests that the seeds of wild soybeans generally contain higher seed antioxidants than those of cultivated soybeans.

The results of germplasm analyses suggest that the reduction of seed antioxidant contents could be a result of soybean domestication and human selection. We have previously constructed an RI population from a wild (W05) and a cultivated (C08) soybean parent, and used this population to identify a number of QTLs of important agronomic traits (Qi et al., 2014). The seed antioxidant content QTL identified in the prior study was based on whole-seed analyses. To reduce the possible interference of anthocyanin from the seed coat in the biochemical assays in this study, we measured the seed antioxidant contents, seed phenolic contents, and seed flavonoid contents using hulled seeds of the RI population (Supplementary Figure S1).

**FIGURE 2:**
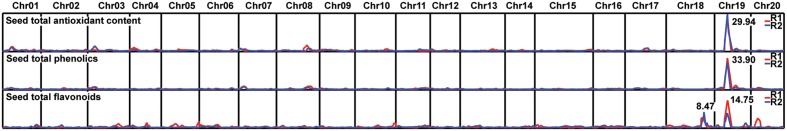
**Log-of-odds (LOD) score distribution of quantitative trait loci (QTLs) across the 20 chromosomes**. The maximum LOD score of each trait is indicated next to the peak. Red and blue lines represent the LOD scores of two biological repeats.

To evaluate the relationships among the contents of total antioxidants, phenolics, and flavonoids in seeds, we performed statistical correlation analyses (**Table 1**). These three parameters exhibit a high positive correlation between each pair of parameters in the comparison. The high correlation among these 3 parameters of seed contents prompted us to further investigate whether they are controlled by the same QTL. By adopting the bin map method (Qi et al., 2014), a major QTL controlling antioxidant contents of hulled seeds was mapped to a 208-kb region on chromosome 19 with a log-of-odds (LOD) score of 29.94, which explained 62% of the variance (**Figure 2**; **Table 2**). The QTL of seed phenolic contents was mapped to the same region with a comparable LOD score of 33.90 and explained 64.27% of the variance (**Figure 2**; **Table 2**). One major QTL (LOD score = 14.75) of seed flavonoid contents was mapped on chromosome 19 spanning a 820-kb region overlapping with the QTLs of seed antioxidant contents and seed phenolic contents. This explained 31.69% of the variance. Furthermore, a second QTL of seed flavonoid contents was mapped to chromosome 18 spanning a 5-Mb genomic region (**Table 2**). Since QTL Cartographer assumes normally distributed phenotypic data, we have run also the same QTL analysis using the data transformed for abnormal distribution. The transformed data also gave the same QTL region on chromosome 19 (Supplementary Table S2 and Supplementary Figure S2).

**Table 1:** Correlation analyses of different traits.

Year	Trait 1	Trait 2	*n*	Pearson		Spearman	
				*r*	*p*-Value	rho	*p*-Value
2010	Seed antioxidant content	Total phenolics	90	0.90	0	0.90	0.0000
	Seed antioxidant content	Total flavonoids	90	0.61	0	0.60	0.0000
	Total phenolics	Total flavonoids	91	0.70	0	0.69	0.0000
2011	Seed antioxidant content	Total phenolics	90	0.95	0	0.96	0.0000
	Seed antioxidant content	Total flavonoids	80	0.45	0	0.30	0.0076
	Total phenolics	Total flavonoids	81	0.71	0	0.32	0.0033

**Table 2:** Major QTLs identified using hulled soybean seeds.

Agronomic traits	LOD cut-off^a^	Chr. no.	Var (%)	QTL position			
				Start Position	End Position	Physical length (kb)	Genetic distance (cM)
Seed antioxidant content	3.9711	19	62.14	37457038	37665887	208.8	1.3
Seed phenolics	4.6642	19	64.27	37457038	37665887	208.8	1.3
Seed flavonoids	3.8153	18	19.27	52716510	58291463	5575.0	12.0
		19	31.69	37315018	38134523	819.5	4.8

*Chr., chromosome; LOD, log-of-odds; Var, variance*.

*^a^LOD score cut-off of major QTLs was determined by permutation tests (1,000 times; *P* < 0.05). The highest values among the two biological replicates were shown*.

### Four Putative MATE Transporter Genes are Found Within the Genomic Region of the QTL for Seed Antioxidant Contents

The QTL region of seed antioxidant contents overlaps with both the QTLs of seed phenolic contents and seed flavonoid contents. It contains 14 annotated gene loci based on the Williams 82 reference genome (Gmax1.01). None of the genes encode functions that are related to the phenylpropanoid pathway of soybean (Reinprecht et al., 2013), the essential metabolic pathway for synthesizing phenolics and flavonoids. However, there are three putative MATE transporter genes found within this QTL region (*Glyma19g29860*, *Glyma19g29870*, and *Glyma19g29940*) and one adjacent to this QTL (*Glyma19g29970*). We named the four putative MATE transporter genes *GmMATE1-4* (Supplementary Table S3).

**FIGURE 3:**
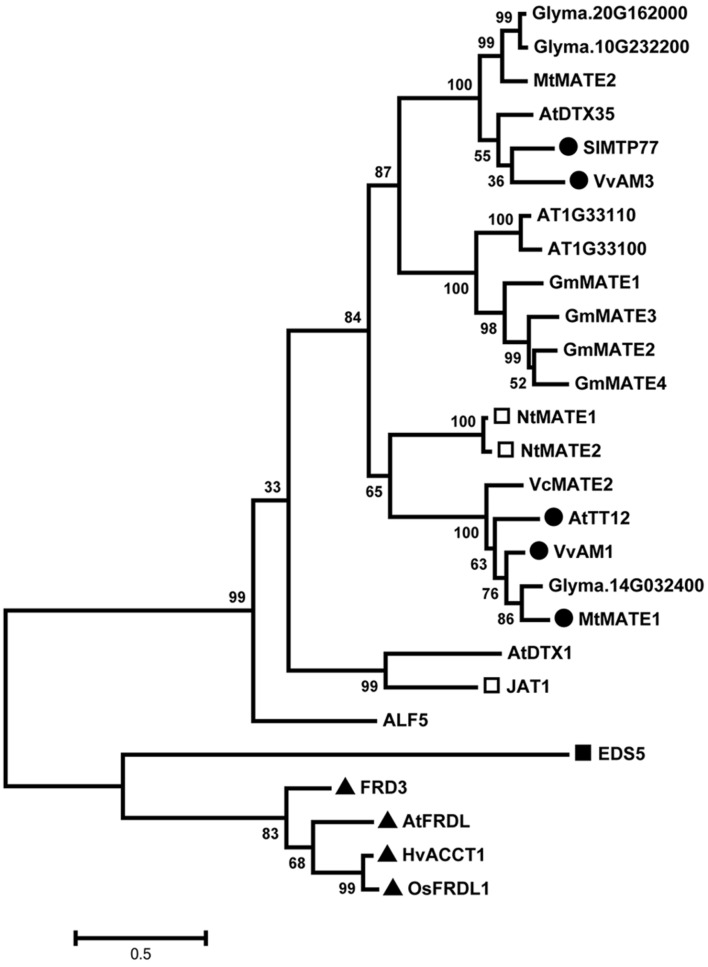
**Phylogenetic study of the 4 multidrug and toxic compound extrusion (MATE) proteins in the antioxidant QTL and selected MATE eﬄux proteins**. The amino acid sequences of selected proteins were aligned using the MEGA6 program. AT1G33110 (62% identities) and AT1G33100 (64% identities) are two closest homologs of GmMATE1 in *Arabidopsis*. Glyma.14G032400 (73% identities) is the closest homolog of AtTT2 in soybean. Glyma.10G232200 (69% identities) and Glyma.20G162000 (69% identities) are two closet homologs of AtDTX35 in soybean. Closed circles mark phenolic-transporting MATE proteins. Open squares mark alkaloid-transporting MATE proteins. Closed squares mark salicylic acid-transporting MATE proteins. Closed triangles mark citrate-transporting MATE proteins. Genbank /NCBI REFSEQ/Locus tag: ALF5: AT3G23560.1; AtDTX1: AT2G04070.1; AtDTX35: AT4G25640.2; AtFRDL: AT1G51340.1; AtTT12: At3g59030.1; EDS5: NP_195614.2; FRD3: CCH27266.1; HvACCT1: BAF75822.1; JAT1: CAQ51477.1; MtMATE1: ACX37118.1; MtMATE2: ADV04045.1; NtMATE1: BAF47751.1; NtMATE2: BAF47752.1; OsFRDL1: BAF11300.2; SlMTP77: NP_001234424.1; VcMATE2: AHH83753.1; VvAM1: XP_002282932.1; VvAM4: NP_001290007.1.

Phylogenetic analyses of the predicted protein sequences of GmMATE1-4 with published MATE transporters of known substrates showed that they cluster with other flavonoid-transporting MATE transporters (**Figure 3**). However, none of the 4 MATE transporters in the antioxidant contents QTL are direct orthologs of known flavonoid-transporting MATE transporters such as AtTT12 and SlMTP77. Instead, GmMATE1-4 forms a distinct clade in the phylogenetic tree.

**FIGURE 4:**
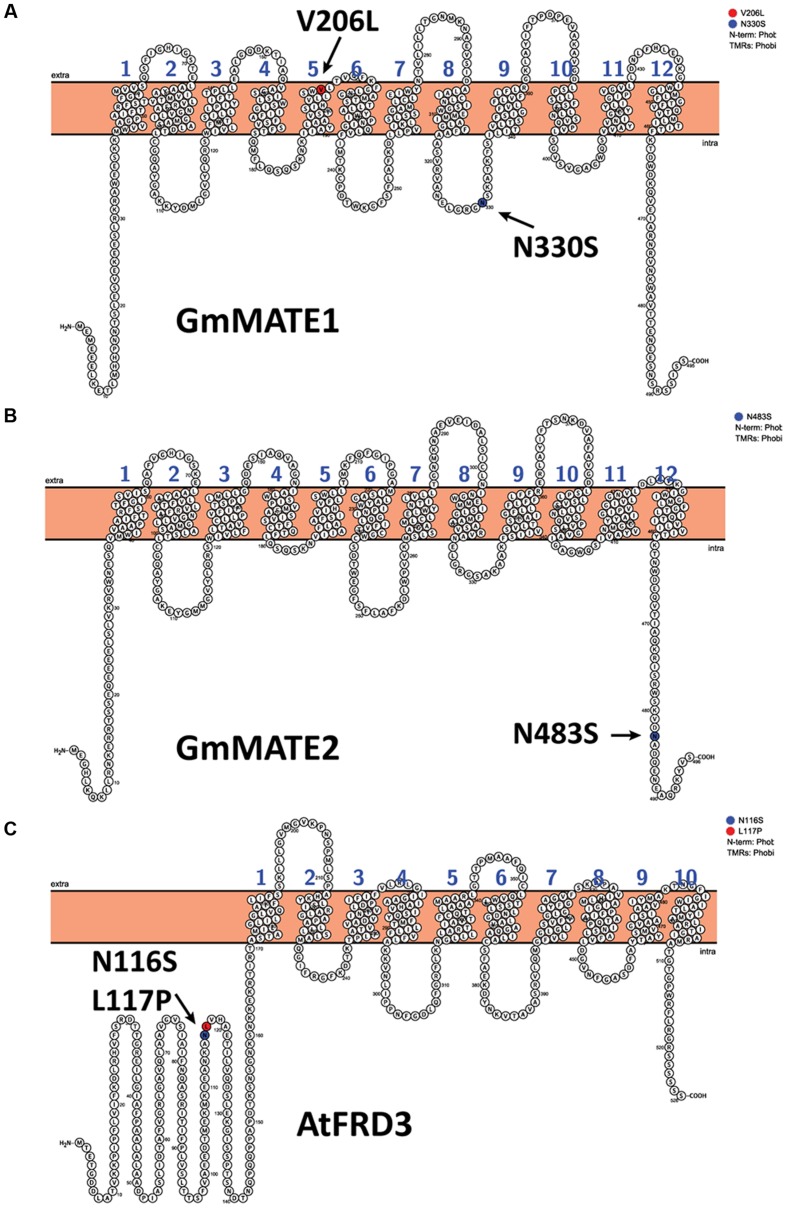
**Transmembrane topologies and non-synonymous amino acids of MATE transporters**. The figure shows the transmembrane topologies of **(A)** GmMATE1 and **(B)** GmMATE2, both from C08, and **(C)** AtFRD3. Non-synonymous changes between C08 and W05 are highlighted and indicated by arrows **(A**,**B)**. The mutations that abrogated the function of AtFRD3 are highlighted and indicated by arrows.

To investigate the possible differences in the gene structures, we successfully amplified the coding sequences of *GmMATE1*, *GmMATE2*, and *GmMATE4* from W05 and C08 cDNAs. Basically, cDNA sequences of C08 resembled those of Williams 82. Between C08 and W05, there are two non-synonymous SNPs in *GmMATE1*: V206L and N330S (**Figure 4A**). In addition, there is one non-synonymous SNP, N483S, in *GmMATE2* (**Figure 4B**). No non-synonymous SNP was found in *GmMATE4*. Full-length coding sequences of *GmMATE1* and *GmMATE2* for W05 have been submitted to GenBank database under accession numbers KX255828 and KX255829, respectively.

**FIGURE 5:**
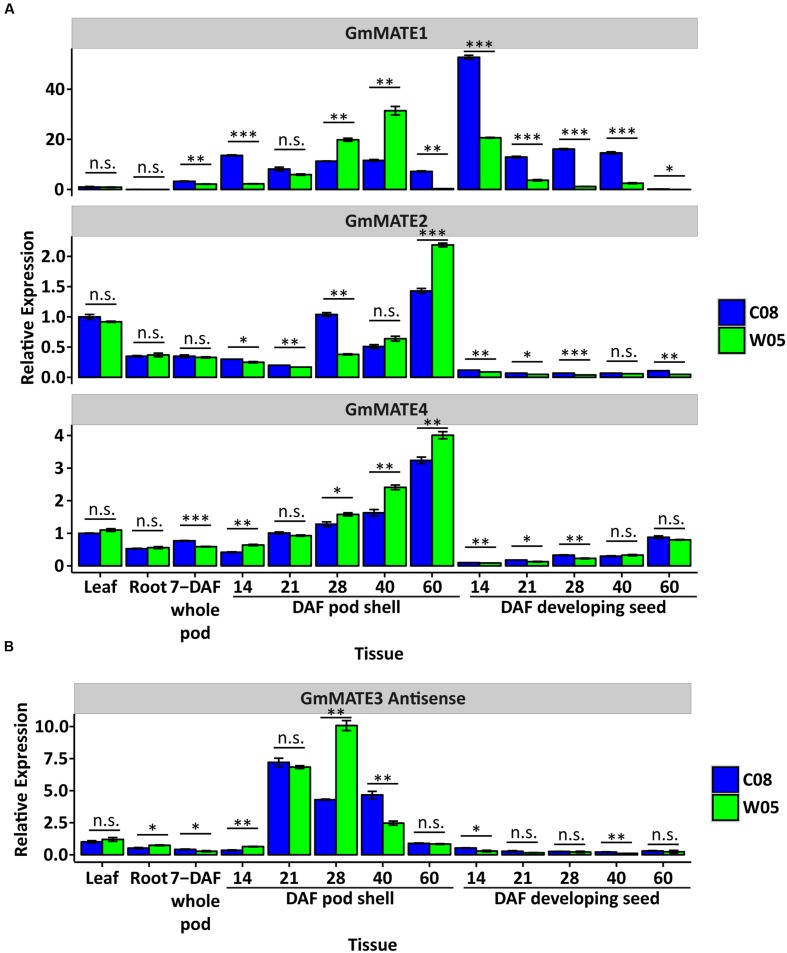
**Expression study of *GmMATE1*, *2*, *4* and antisense transcript levels of *GmMATE3* in different tissues**. **(A)** Expression of *GmMATE1*, *2* and *4*. **(B)** Expression of the antisense transcripts of *GmMATE3*. The expression level of each target gene in C08 leaf was set to 1 as the basis for comparison, after normalizing with the housekeeping genes, *ELF1b* and *Bic-C2*, using the 2^-ΔΔC_T_^ method. DAF: days after flowering. *N* = 3. Error bar: ± s.e.m. Expression of target gene in different tissue was compared between C08 and W05 using Student’s *t*-test. n.s.: not significant; ^∗^*p* < 0.05; ^∗∗^*p* < 0.01; ^∗∗∗^*p* < 0.001.

To determine if there are any differences in the 4 *MATE* genes from W05 versus C08, expression studies and sequence analyses were performed. RT-qPCR studies showed that *GmMATE1* expression is up-regulated in the pod and seed (**Figure 5A**). *GmMATE2* is generally expressed in all tissue types except in the seed (**Figure 5A**). *GmMATE4* is expressed in all tissue types, but with higher expression levels in old pods (**Figure 5A**).

### Expression of Antisense *GmMATE3* Transcripts in Pods

We were not able to amplify the *GmMATE3* coding sequence (1080 bp) based on the gene model *Glyma19g29940* using cDNAs from different tissues. On the other hand, PCR products of a larger than expected size were amplified from the cDNAs of 28-DAF or older pod shells. We further confirmed the orientation of this PCR product by amplifying it from cDNAs synthesized using a *GmMATE3* forward primer for antisense transcripts or cDNAs synthesized using a reverse primer for sense transcripts. Sequencing results confirmed that the PCR product is the antisense transcript of *GmMATE3* (Supplementary Figure S3). The expression of *GmMATE3* antisense transcripts increased around 28 days after flowering (**Figure 5B**). While the natural antisense transcripts (NAT) of *GmMATE3* may regulate the expression of other *MATE* genes, the expression levels of three other *MATE* genes, which share high sequence similarities with *GmMATE3*, were not associated with the expression of the antisense transcripts of *GmMATE3* (Supplementary Figure S4).

## Discussion

Investigation of 30 germplasms shows that wild soybean seeds generally have higher antioxidant contents compared to cultivated soybeans. It is possible that the reduction in antioxidant contents in soybean seeds was one unintended consequence of human selection during the domestication process. Breeders tended to select traits such as seed coat color, mature pod color, seed germination rate, yield, and so on. It is possible that a locus controlling seed antioxidant contents in wild soybean is linked to unfavorable traits, or that the accumulation of antioxidants or phenolic compounds in seeds is closely linked to certain unfavorable traits that were selected against. One possibility is that the accumulation of phenolics leads to increased seed coat hardness and hence lowers the germination rate in newly collected seeds (Zhou et al., 2010). Another possible explanation is that the high antioxidant contents in seeds seem to be associated with the browning of mature pods (Qi et al., 2014). The dirty appearance of the brown pod may also be an unfavorable trait during selection.

We have previously demonstrated that seed antioxidant contents were not associated with the seed coat color or the seed anthocyanin content in an RI population originated from a wild (W05) and a cultivated soybean (C08) (Qi et al., 2014). Using the same RI population, we further our study using hulled soybeans. As expected, the QTLs of total phenolics and total flavonoids were mapped to the same region on chromosome 19 as the antioxidant content QTL. The result was consistent with the fact that the total phenolics and total flavonoids showed significant correlations with the total antioxidant content. Because of this relationship, we believed that phenolics and flavonoids in the seed may account for most of the antioxidant activities as previously suggested (Malencic et al., 2007).Since the major phenolic metabolites accumulated in soybean seeds are isoflavonoids (Seo and Morr, 1984), there have been a number of studies mapping loci controlling isoflavone contents, using genetic populations derived from cultivated soybeans (Primomo et al., 2005; Zeng et al., 2009; Gutierrez-Gonzalez et al., 2010b; Wang et al., 2015; Zhao et al., 2015). These mapped loci do not overlap with the QTL on chromosome 19 identified in this work. Our use of an RI population derived from both a wild parent and a cultivated one has enabled the discovery of new QTLs.

While we believe that the lower antioxidant content in cultivated soybean seeds is related to domestication, a resequencing project involving 302 wild and cultivated soybean accessions has identified a selective signal in soybean improvement on chromosome 19 spanning 36,860,001-37,570,000 (Zhou et al., 2015), which partially overlaps with our antioxidant QTL (37,457,038-37,665,887). The cross-population composite likelihood ratio of this signal is 36.23 (threshold = 4.6), which was the 4th highest among the 109 regions identified (Zhou et al., 2015). It is interesting that this region only appears to be a selective signal for improvement but not domestication (Zhou et al., 2015), suggesting that this feature may have been bottlenecked during the soybean improvement process. Currently we cannot rule out the possibility that the antioxidant QTL from wild soybean is linked to a detrimental locus. Therefore, our results still have the potential to improve the antioxidant and phenolic compounds content in cultivated soybean through a strategic breeding process.

Within the 208-kb antioxidant QTL locus, there are about 14 gene loci in the Williams 82 reference genome. None of these were predicted as part of the phenylpropanoid pathway (Reinprecht et al., 2013), which corresponds to the biosynthesis of flavonoids. Instead, there are MATE protein-encoding genes located within, or in close proximity to, this QTL region. MATE transporters have been found to mediate the transportation of various molecules in plant, ranging from metal ions to biomolecules. Some of them are involved in phenolics transportation. For example, AtTT12 from *Arabidopsis* and MtMATE1 from Medicago are vacuolar flavonoid/H^+^ antiporter, preferentially transporting epicatechin 3′-O-glucoside for proanthocyanidin biosynthesis in the seed coat (Marinova et al., 2007; Zhao and Dixon, 2009). SlMTP77 from tomato, MtMATE2 from Medicago and VvAM1 and anthoVvAM3 from grapevine are involved in anthocyanin transportation (Mathews et al., 2003; Gomez et al., 2009; Zhao et al., 2011). In our phylogenetic study here using other published MATE proteins, GmMATE1-4 are clustered with flavonoid-transporting MATE proteins, suggesting that they may transport flavonoid-related compounds.

While there is no known phenolic compound or flavonoid biosynthetic genes involved in the antioxidant locus, the presence of the four MATE protein-encoding genes infers that soybean seed antioxidant contents may not be limited by biosynthesis but by transportation or accumulation. On the other hand, in our previous study, we have discovered that the seed antioxidant content is correlated with the mature pod color (Qi et al., 2014). It has been reported that many secondary plant compounds are transported from their sites of synthesis to the sites of accumulation (Dhaubhadel, 2011). It has been demonstrated that although the soybean embryo has the ability to synthesize flavonoids, the synthesis in maternal tissues may contribute significantly to the total accumulation in sink organs such as seeds at maturity (Dhaubhadel et al., 2003). However, how flavonoids or their precursors are transported between tissues remains unclear. Interestingly, our MATE transporters expression data support the idea that they could be involved in flavonoids or precursors transportation between tissues. Expression patterns of *GmMATE1*, *2*, and *4* show high expression levels at 60 DAF with a matching gradual increase during pod development and seed filling.

A previous study suggested that two non-synonymous amino acid changes (N116S and L117P) are sufficient to abrogate citrate transportation by a MATE transporter AtFRD3 (Pineau et al., 2012) (**Figure 4C**). Interestingly, a similar type of substitution can be found in GmMATE1 (N330S) and GmMATE2 (N483S) (**Figures 4A,B**). Plants have a large number of MATE genes, in contrast to a relatively small number in bacteria and animals (Omote et al., 2006). It is fascinating why these transporters have diversified in plants and why they are able to transport a large variety of compounds. In this context, based on our results and previous studies (Pineau et al., 2012), we speculate that amino acid substitutions could contribute to the changes in protein activities or transport specificities.

A NAT of *GmMATE3* was detected during pod maturation (**Figure 5B**; Supplementary Figure S3). The expression of this NAT coincided with the timing of the accumulation of isoflavones in developing seeds (∼30 DAF) as previously reported (Gutierrez-Gonzalez et al., 2010a). NATs have been widely detected and studied in different organisms (Morris, 2012; Wight and Werner, 2013). One of the well-studied functions of NAT is to regulate the expression in *cis* and *trans*. However, the expression of the NAT of *GmMATE3* was not associated with the expression of any of the 6 *MATE* genes analyzed in this study (**Figure 5**; Supplementary Figure S4). On the other hand, it is possible that the NAT of *GmMATE3* may regulate the functions of MATE transporters through other mechanisms such as RNA editing, inhibition of translation, inhibition of RNA export from the nucleus, etc.

## Conclusion

Quantitative trait loci corresponding to high antioxidant contents, high total phenolics and high total flavonoids in the wild soybean, W05, were mapped to the same position on chromosome 19. This genomic region may be linked to domestication or the improvement of soybean. Hence, more efforts will be needed to investigate the potential of using this QTL to help improve the antioxidant contents in cultivated soybean. On the other hand, 4 MATE transporter-encoding genes were found within and adjacent to this locus, suggesting that the high antioxidant content in wild soybean seeds could be related to intercellular phenolic compounds transportation. Non-synonymous changes were identified in *GmMATE1* and *GmMATE2*, while *GmMATE3* encodes an antisense transcript that is expressed in pods. Whether the polymorphisms in GmMATE proteins are a major determinant of the antioxidant contents, and whether the antisense transcript of *GmMATE3* plays an important regulatory role, await further functional investigations.

## Author Contributions

M-WL, NBM, and H-ML designed the experiments, wrote the manuscript and analyzed the data. M-WL, and XQ performed the bioinformatics analysis and F-LW collected field data. NBM, C-FW, K-SW, and JW-HW performed biochemical assays and M-WL, K-PL, and M-SN carried out the molecular experiments.

## Conflict of Interest Statement

The authors declare that the research was conducted in the absence of any commercial or financial relationships that could be construed as a potential conflict of interest.

## Footnotes

1 http://statgen.ncsu.edu/qtlcart
