# Supplementary material for: QTLs Regulating the Contents of Antioxidants, Phenolics, and Flavonoids in Soybean Seeds Share a Common Genomic Region
Source: Front Plant Sci. 2016 Jun 14;7:854. doi: 10.3389/fpls.2016.00854 (PMC4906965; doi:10.3389/fpls.2016.00854)
Supplement: Supplementary file 1 [file Data_Sheet_1.PDF]

QTLs regulating the contents of antioxidants, phenolics and flavonoids in soybean seeds share a common genomic region

Man-Wah Li[†,a], Nacira Belen Muñoz[†,a,b,c], Chi-Fai Wong[a], Fuk-Ling Wong[a], Kwong-Sen Wong[a], Johanna Wing-Hang Wong[a], Xinpeng Qi[a], Kwan-Pok Li[a], Ming-Sin Ng[a], Hon-Ming Lam[*,a]

Supplementary Information

Supplementary Table S1. Mature pod color, seed coat color of 30 soybean germplasms

Germplasm		Mature pod color *	Seed coat color
C01	Cultivated	Grayish brown	yellow
C02	Cultivated	Dark brown	yellow
C08	Cultivated	Tan	yellow
C12	Cultivated	Tan	yellow
C14	Cultivated	Tan	yellow
C16	Cultivated	Tan	yellow
C17	Cultivated	Dark brown	yellow
C19	Cultivated	Tan	yellow
C24	Cultivated	Brown	greenish yellow
C27	Cultivated	Tan	yellow
C30	Cultivated	Brown	yellow
C33	Cultivated	Tan	yellow
C34	Cultivated	Tan	yellow
C35	Cultivated	Tan	yellow
W01	Wild	Tan	black
W02	Wild	Tan	black
W03	Wild	Brown	black
W04	Wild	Tan	brown
W05	Wild	Dark brown	black
W06	Wild	Grayish brown	black
W07	Wild	Dark brown	black
W08	Wild	Brown	black
W09	Wild	Brown	black
W10	Wild	Black	brown
W11	Wild	Brown	black
W13	Wild	Tan	black
W14	Wild	Grayish brown	black
W15	Wild	Grayish brown	brown
W16	Wild	Dark brown	black
W17	Wild	Black	black

Supplementary Table S2. Major QTL identified using transformed data. Only QTL consistent in two replicates were shown.

Agronomic traits	LOD cutoff *	Chr. no.	Var (%)	QTL position			
				Start Position	End Position	Physical length (kb)	Genetic distance (cM)
Seed antioxidant content	3.9116	19	66.02	37457038	37665887	208849	1.3
Seed phenolics	3.9189	19	62.38	37457038	37665887	208849	1.3
Seed flavonoids	3.9130	19	28.81	37315018	37665887	350869	3.2

Chr., chromosome; LOD, log-of-odds; Var, variance.

*LOD score cut-off of major QTLs was determined by permutation tests (1,000 times; $P<0.05$). The highest values among the two biological replicates were shown.

Supplementary Table S3. Information of primers used in this study.

Gene	Gene ID	*Primer Sequence 5' -> 3'	Primer Description
Bic-C2	Glyma.03G064800	GCCTTAATGATGTGAATGGT	Forward primer for RT-qPCR
		AGAGATCATGTTCCCACTTG	Reverse primer for RT-qPCR
ELF1b	Glyma.02G276600 & Glyma.14G039100	CCACTGCTGAAGAAGATGATGATG	Forward primer for RT-qPCR
		AAGGACAGAAGACTTGCCACTC	Reverse primer for RT-qPCR
GmMATE1	Glyma19g29860 Glyma.19G120200	AATCTAGAATGGAAATGGAAGAGGAG	Forward primer for the amplification of full length cDNA
		AACTCGAGCTAGCTAGATATGCTTGA	Reverse primer for the amplification of full length cDNA
		CATTCACCAGCCAAATGTTC	Forward primer for RT-qPCR
		TTGAGCCCAAACTTGAACTG	Reverse primer for RT-qPCR
GmMATE2	Glyma19g29870 Glyma.19G120300	ATGGAGGGGAATCTAGAGAAGAAGCT	Forward primer for the amplification of full length cDNA
		CTAATTATCTGATGTAGTTGTTTCATGATC	Reverse primer for the amplification of full length cDNA
		CAATGGATGGGAAATGATGA	Forward primer for RT-qPCR
		AACAGAAGGAATCCAATGGC	Reverse primer for RT-qPCR
GmMATE3	Glyma19g29940 Glyma.19G120700	ATGTCAAGTGCGCTGTCAACACTAT	Forward primer for the amplification of full length cDNA
		TCAAATACAAGTATGCAACCTGAGA	Reverse primer for the amplification of full length cDNA
		CTTGGCGGCTTTCTCAATAG	Forward primer for RT-qPCR (exon 1)
		AGGAATCCAGAATGCCAAAC	Reverse primer for RT-qPCR (exon 1)
GmMATE4	Glyma19g29970 Glyma.19G120900	ATGGAGGGGAATCTAGAGAAGAAGCT	Forward primer for the amplification of full length cDNA
		CTAATTATCTGATGTAGTTGTTTCATGATC	Reverse primer for the amplification of full length cDNA
		GATAGCATTTGGTTTCATGGC	Forward primer for RT-qPCR
		TTTGGAGCTTCCTCTTCCAA	Reverse primer for RT-qPCR
Glyma.03G005600	Glyma.03G005600	TGGATTGGAATGTTGTTTGG	Forward primer for RT-

			qPCR
		CAGATTCCACCTTGGACCAC	Reverse primer for RT-qPCR
Glyma.03G005400	Glyma.03G005400	GATAGCACTTGGTTTCATGGC	Forward primer for RT-qPCR
		GCAGCTTGTGAGCTTCCTCT	Reverse primer for RT-qPCR
Glyma.03G005800	Glyma.03G005800	GGATTTGCAAGTCAAGGGAA	Forward primer for RT-qPCR
		AGCAATGGTAACCTGCTCGT	Reverse primer for RT-qPCR

* Underlined are extra sequences for cloning purpose

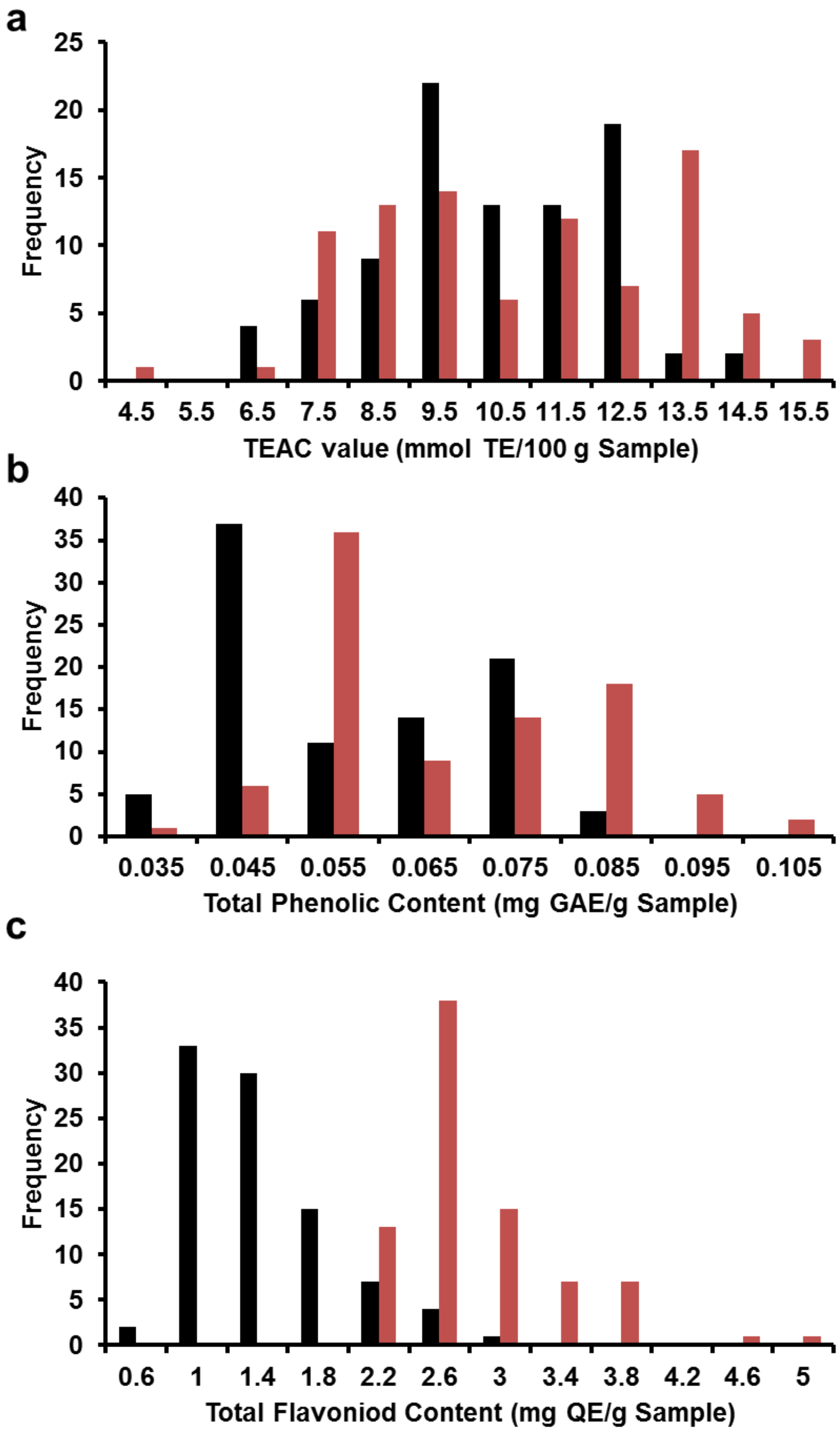

Supplementary Figure S1. Distribution of raw data used for QTL mapping. (a) Seed total antioxidant content, (b) Seed total phenolics, (c) Seed total flavonoid content. Black and red bars represent data of two biological repeats.

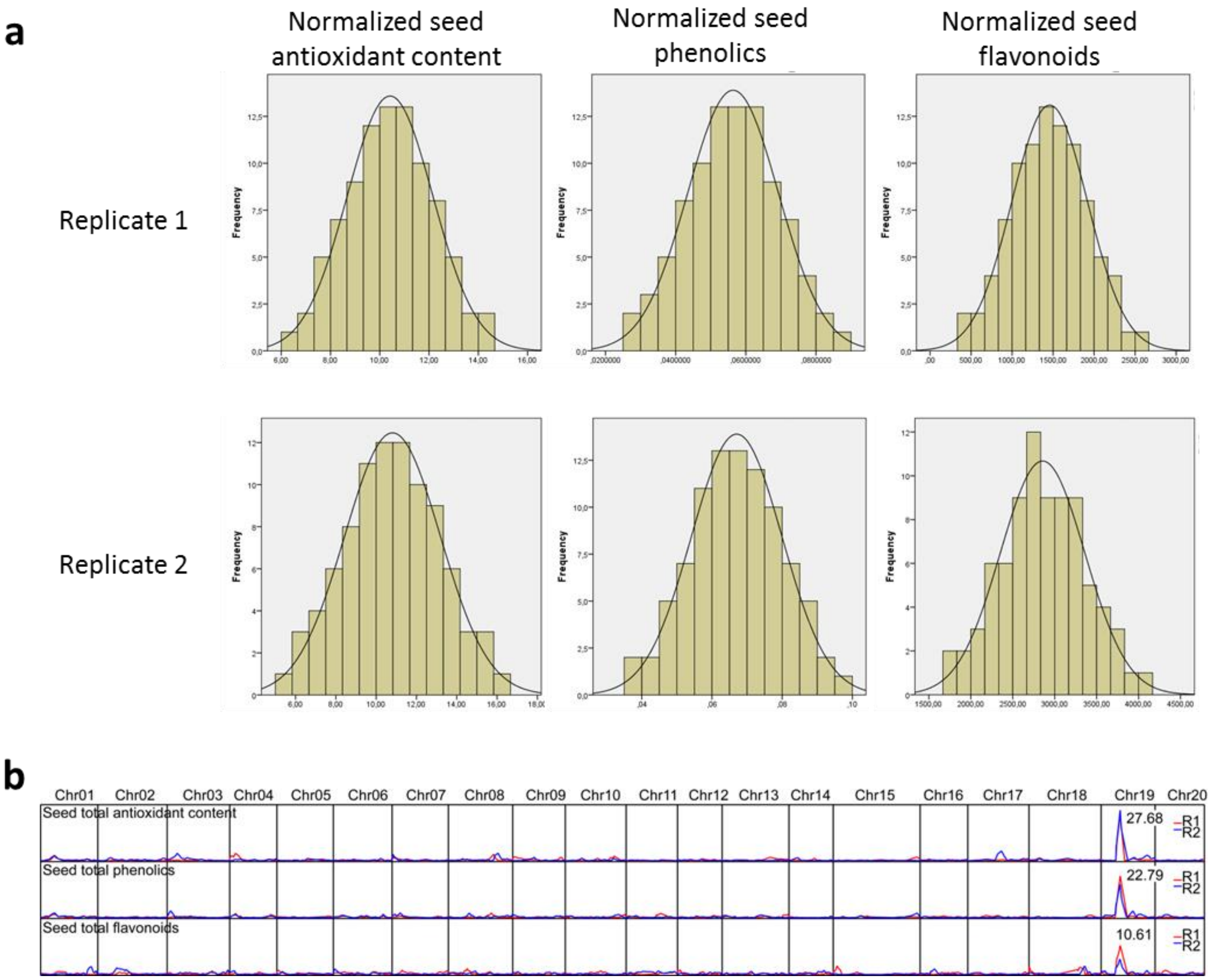

Supplementary Figure S2. QTL analysis using transformed data. (a) Data distribution after transformation. (b) LOD score distribution of QTLs across the 20 chromosomes. Maximum LOD score of each trait was indicated next to the peak. Red and blue lines represent LOD scores of two biological repeats.

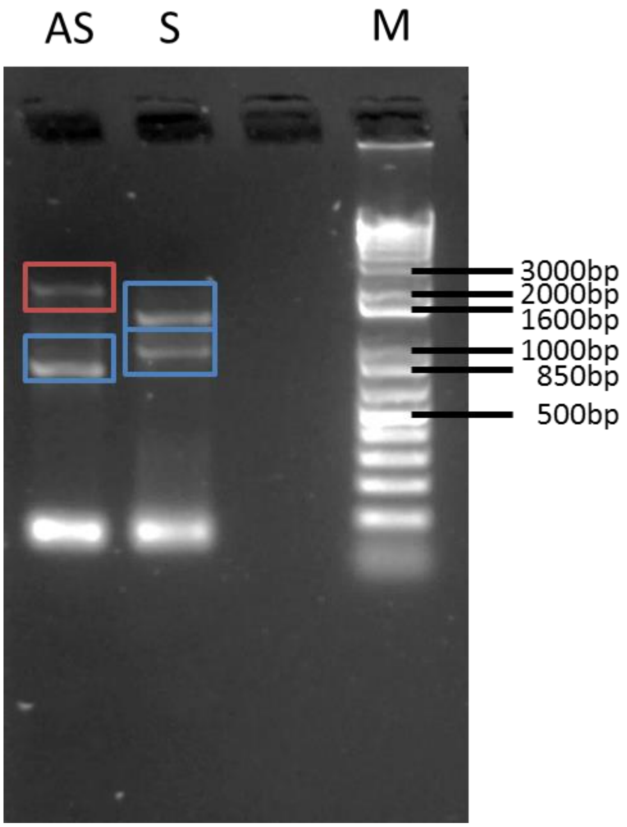

Supplementary Figure S3. Amplification of an antisense *GmMATE3* transcript. Amplification of *GmMATE3* from cDNA originated from 28-DAF pod shell. Red box indicated specific amplification bands for the antisense transcript of *GmMATE3*. Blue boxes indicated non-specific bands of MATEs from other loci. M: 1kb plus DNA ladder; AS: PCR product from cDNA produced using full length forward primer for antisense transcript; S: PCR product from cDNA produced using full length reverse primer for sense transcript.

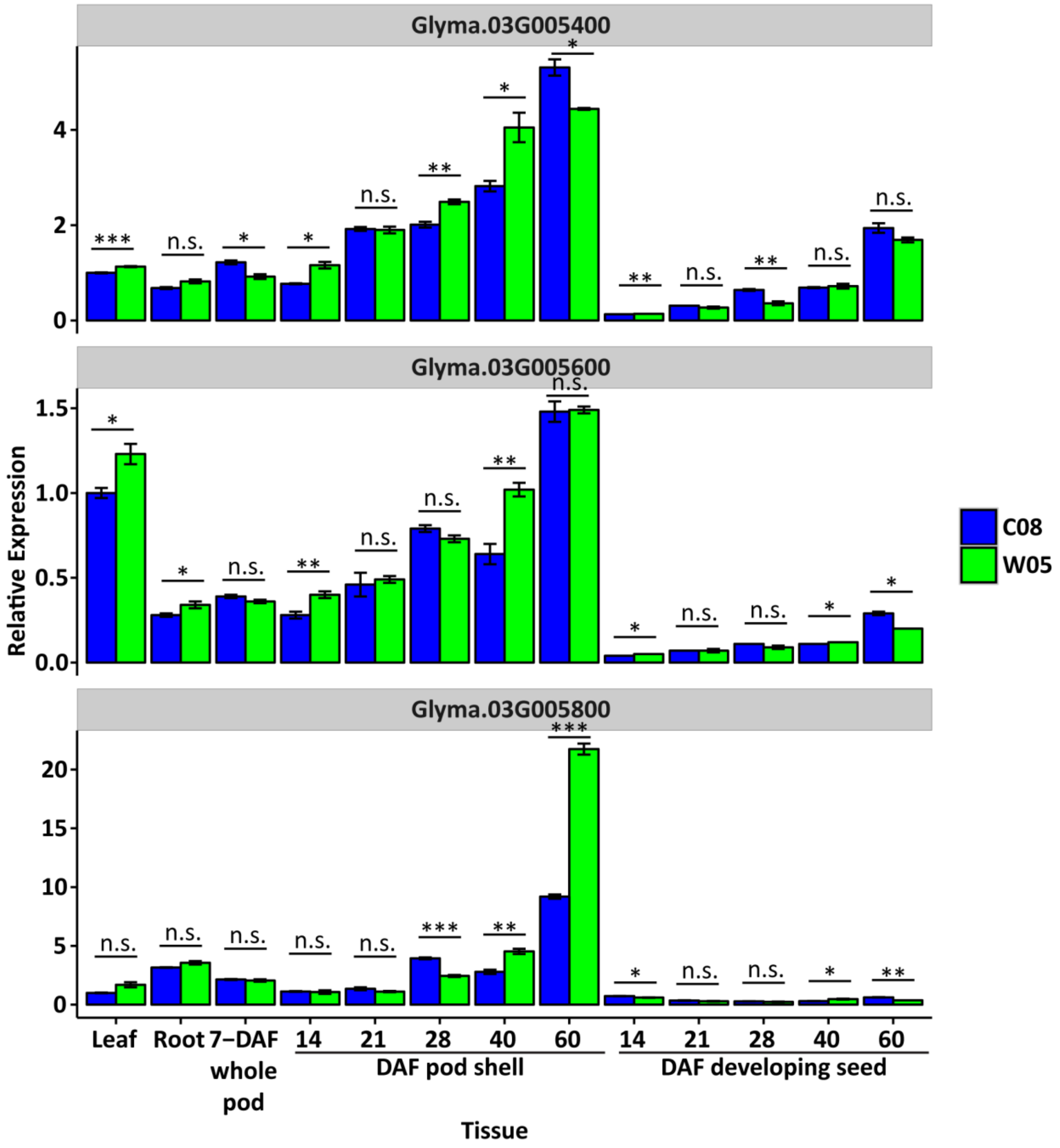

Supplementary Figure S4. Expression study of 3 additional MATE genes in different tissues. Expression level of target gene in C08 Leaf was set as 1 for comparison. DAF: days after flowering. N = 3. Error bar: ± s.e.m. Expression of target gene in different tissue was compared between C08 and W05 using Student's t-test. n.s.: not significant; *: $p<0.05$; **: $p<0.01$; ***: $p<0.001$.
